# Construction and Applicability Scenarios of 3D Neurovascular Unit Models In Vitro

**DOI:** 10.3390/biom16091250

**Published:** 2026-08-28

**Authors:** Baojian Yu, Zekai Shao, Zhuona Ni, Yuxin Gao, Ziyang Ding, Weifeng Jiang, Lin Li, Lisheng Chu

**Affiliations:** 1 Department of Physiology, Zhejiang Chinese Medical University, Hangzhou 310053, China; 2240032@wyuas.edu.cn (B.Y.); 202411011411016@zcmu.edu.cn (Z.N.); 202311115911066@zcmu.edu.cn (Z.D.); 202111011411031@zcmu.edu.cn (W.J.); 2 Department of Traditional Dai-Thai Medicine, West Yunnan University of Applied Sciences, Xishuangbanna 666100, China; 3 Department of Public Health, Zhejiang Chinese Medical University, Hangzhou 310053, China; 202312213603021@zcmu.edu.cn; 4 Department of Rehabilitation Medicine, Zhejiang Chinese Medical University, Hangzhou 310053, China; 202312214403005@zcmu.edu.cn

**Keywords:** neurovascular unit, three-dimensional models, in vitro techniques, blood-brain barrier, cell culture techniques, central nervous system diseases, tissue engineering

## Abstract

The neurovascular unit (NVU) is composed of a diverse array of cells and an extracellular matrix (ECM). Neural cells and blood vessels are intricately interconnected, forming a cohesive whole. Specific cellular components and structures within the NVU play an indispensable role in maintaining homeostasis of the central nervous system (CNS). With the advancement and maturation of cell co-culture technology, various three-dimensional (3D) NVU models continue to emerge, offering a more objective and comprehensive perspective for in vitro studies of CNS diseases. Specifically, these 3D NVU models include Transwell Chamber models, gel-polydimethylsiloxane (PDMS)-based 3D models, self-assembled NVU models and microfluidic NVU models, which reconstruct the complex NVU architecture to varying degrees. This review systematically summarizes multiple 3D construction strategies for in vitro NVU to overcome the limitations of conventional cellular tests or animal experiments, highlights the critical roles of biomimetic gel in recapitulating native cell-gel crosstalk, comparatively analyzes four major 3D NVU technical routes in terms of cellular composition, vascular morphology, barrier performance, and reproducibility, categorizes application scenarios of 3D NVU platforms oriented to practical research demands, including oxygen-glucose deprivation/reoxygenation (OGD/R) injury modeling, blood-brain barrier (BBB) permeability assay, CNS drug penetration screening, neuroinflammation and neurotoxicity evaluation, proposes practical principles for model selection under different experimental purposes, and concludes with current bottlenecks, including imperfect vascular network maturation and lack of unified evaluation criteria, together with future perspectives for standardized 3D NVU in vitro. By comparing the advantages and limitations of these approaches, we aim to clarify their optimal applicability for investigating specific pathological mechanisms and screening potential therapeutics.

## 1. Introduction

The concept of the neurovascular unit (NVU) was introduced during the 2001 Stroke Progress Review Group Meeting of the National Institute of Neurological Disorders and Stroke [1]. It aimed to revolutionize stroke treatment strategies: focusing research away from individual cell types and isolated cellular signaling pathways and toward holistic regimens integrating varied cell populations, multifactorial components and combinatorial therapies. Furthermore, it advocated a transition from studies at the cellular level to higher organizational levels, even at the sub-organ level, thereby emphasizing the importance of integrative research in stroke prevention and control. Additionally, the concept of the NVU emphasized the interdependence between capillaries, nerve cells, and nerve glial cells, and highlighted the need for a multidimensional perspective to understand the role of nerve cells and brain microvessels in the onset, progression, and prognosis of ischemic stroke.

The NVU is composed of multiple cell types and components, including brain microvascular endothelial cells (BMECs), pericytes (PCs), astrocytes (ACs), microglia (MG), oligodendrocytes (OGs), and neurons, as well as non-cellular elements like the extracellular matrix (ECM) [1]. The neural stem cells (NSCs) did not originally belong to the NVU group, but some NVU models were constructed using them (All NSC-containing models, see Supplementary Table S1).

**Figure 1:**
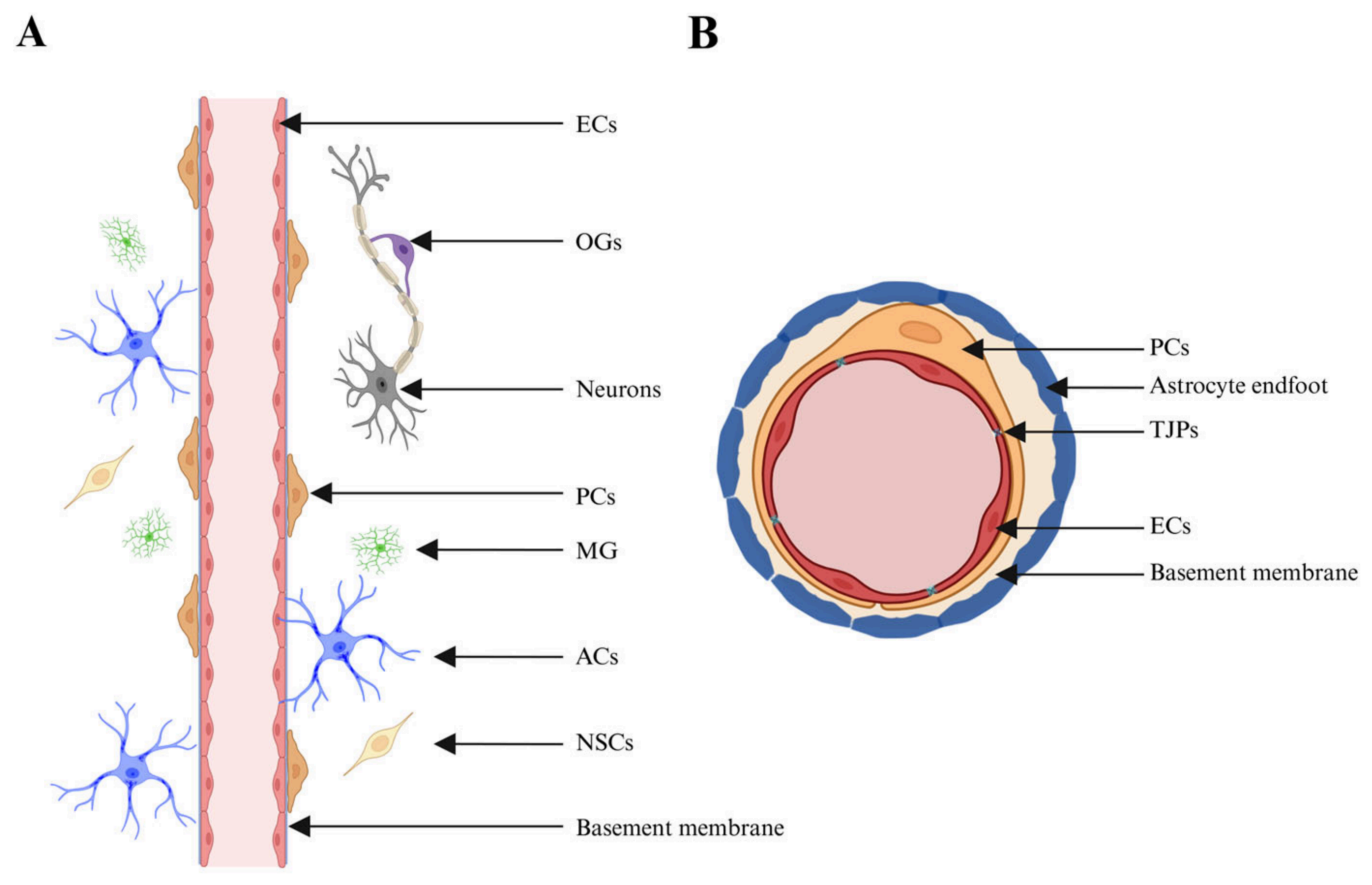
The constituents of the NVU and BBB. (**A**) Longitudinal view illustrating the complex interactions between the brain microvasculature and the surrounding neural parenchyma. The capillary is composed of BMECs anchored to the basement membrane, with PCs embedded within it. The unit is further integrated with elements, including ACs, neurons, MG, OGs, and NSCs, forming a functional microenvironment. (**B**) Cross-sectional view detailing the architecture of the BBB. The lumen is lined by a continuous layer of BMECs connected by TJPs. This inner layer is encircled by the basement membrane containing PCs and is almost entirely enveloped by the perivascular astrocyte endfeet, highlighting the stratified barrier structure. Created in BioRender. Gao, Y. (2026) https://BioRender.com/6ok7lgw.

Therefore, for the sake of convenience in summarization, this article also regards NSCs as components of the NVU group (Figure 1A). Structurally, BMECs form the continuous lining of blood vessels, PCs tightly adhere to the vessel wall, and ACs extend their endfeet to ensheathe the vasculature. These three cell types collaborate to establish the blood-brain barrier (BBB) (Figure 1B), a compact, semipermeable interface enriched with tight junction proteins (TJPs), which is critical for maintaining brain homeostasis [2]. As resident immune cells of the central nervous system (CNS), MG are ramified under physiological resting conditions and perform central immune surveillance. During embryonic development, they eliminate superfluous neurons and prune synapses to participate in the developmental remodeling of neural tissue. Upon tissue injury or inflammation, MG become amoeboid and phagocytose apoptotic cells, tissue debris and pathogens. They exert dual functions: they facilitate local tissue repair and nerve regeneration after injury, yet excessive activation under chronic pathological conditions triggers abundant pro-inflammatory cytokine release, thereby accelerating the progression of neurodegenerative diseases (NDDs) [3,4]. OGs coil around the axons of neurons, forming a myelin sheath providing a foundation for the saltatory conduction of nerve fibers and thereby accelerating the speed of impulse transmission. In addition, they secrete nutritional factors to nourish the axons, the myelin serves to insulate and mechanically protect the axons. While neurons primarily receive stimuli, integrate information, and propagate nerve impulses, a small subset of specialized subtypes participate in consciousness regulation and the modulation of physiological rhythms as their secondary functions, which lack the capacity to nourish other cells. NSCs can be activated to regenerate the structure and function of brain tissue in response to brain damage. Multiple cell populations interact functionally to keep the CNS in homeostasis.

The NVU component cells are located within the ECM constituting the direct microenvironment. The ECM is a critical environmental factor that directly supports cell survival and growth. It comprises a variety of biopolymeric macromolecules, including proteins, glycoproteins, and protein proteoglycans, which facilitate strong cell adhesion, integration, and the formation of structured tissues [5]. The ECM plays a pivotal role in establishing and sustaining distinct microenvironments within tissues by regulating factors such as growth factor receptor/ligand interactions, molecular hydration levels, and microenvironmental acidity and alkalinity [6,7].

Many CNS diseases are associated with disorders of the NVU. Numerous studies concerning the NVU have been published, yet most previous investigations relied on animal models or single-type cell in vitro assays [8,9]. As is well-known, animal experiments are severely limited by ethical concerns, low throughput, and high postoperative mortality [10,11,12]. Conversely, traditional cellular experiments cannot adequately reflect the overall function and three-dimensional (3D) structure of the NVU [13]. Therefore, the use of multi-cell and multi-component NVU 3D models in vitro to replace live animal or monoculture experiments has become an inevitable trend in the study of CNS diseases, laying a preliminary foundation for the exploration of transplantation therapy for brain diseases [14].

Despite the abundance of existing models, a systematic summary regarding their specific selection strategies remains limited. Therefore, this review aims to classify current 3D NVU models and critically evaluate their applicability scenarios, providing a rationale to select the most appropriate model for distinct research purposes.

## 2. The Cellular Composition and Functions of NVU

### 2.1. Functional Comparison of Each Cell in NVU

NVU component cells include BMECs, PCs, ACs, MG, OGs, neurons, and NSCs. Each cell plays its own role (Table 1) and jointly maintains the functional and structural stability of NVU.

### 2.2. The Role of BMECs in NVU

The BMECs are the main cellular components of the BBB, forming capillaries that are surrounded by neurons and glial cells. CNS homeostasis is maintained by the collaboration between various cells [2]. Unlike peripheral capillary endothelial cells (ECs), BMECs are highly enriched with TJPs, facilitating the formation of continuous capillaries and a dense barrier for substance exchange between the systemic circulation and brain tissue [15,16].

Furthermore, single-cell transcriptomic analysis has revealed that BMECs exhibit significant molecular heterogeneity across different brain regions, a feature that is essential for maintaining local CNS homeostasis [17]. Beyond the physical barrier, current studies emphasize that BMECs act as active signaling hubs, they regulate BBB integrity by suppressing transcytosis and communicating with PCs via angiocrine factors [18]. In the development of 3D NVU in vitro models, incorporating these multicellular interactions is now considered the ‘gold standard’ for recapitulating the functional NVU [19].

The BBB is a highly intricate physiological barrier within the body. It rigorously controls the exchange of substances between the brain and the circulatory system, characterized by well-developed TJPs with low permeability and high resistance [20,21]. Additionally, the BBB serves as a safeguard against peripheral pathogens, circulating immune cells, and neurotoxic drugs infiltrating the CNS [22]. On one hand, the distinctive characteristics of the BBB shield the CNS from participation in peripheral cellular and humoral immunity, greatly contributing to CNS protection. On the other hand, these stringent controls on substance exchange make it challenging for many drugs to access the CNS and exert their effects. The structural and functional impairments of the BBB manifest concretely in various CNS diseases such as Alzheimer’s disease (AD) and Parkinson’s disease (PD) [22].

### 2.3. Roles of ACs in NVU

Brain vessels are ensconced by PCs and ACs. ACs represent a subtype of neuroglial cells, bearing similarities to fibroblasts found in peripheral tissues. They establish connections with blood vessels through specialized endfeet, which exhibit remarkable adaptability due to their increased rough endoplasmic reticulum and smooth endoplasmic reticulum, and the presence of the Golgi complex. This complex organelle network supports the polarized trafficking of Aquaporin-4 (AQP4) to the endfeet membrane, a process essential for maintaining the BBB and glymphatic clearance [23].

These characteristics are likely associated with processes involving biological synthesis, membrane maturation, and protein secretion [24]. The endfeet of ACs are enriched with G-protein-coupled receptor (GPCR)-mediated calcium ion (Ca^2+^) channels, facilitating communication with blood vessels through various signaling pathways [25,26,27]. Conversely, Ca^2+^ signals are influenced by plasma osmotic pressure and blood flow pressure [28,29,30]. Additionally, the sub-terminal regions of ACs harbor glutamate transporters, sodium-calcium exchangers, and sodium-potassium pumps, whereas connexin43 together with these ion transporters are concentrated at AC branch endings [31,32]. These compartments maintain abundant glucose reserves to meet the lifelong energetic demands of ACs [31,32]. Both the lobules and branches enable ACs to establish connections with synapses of neurons. In the cortex, approximately 60 percent of synapses interface with ACs lobules, while the remaining 40 percent engage with ACs branches [33]. Research indicates that vascular endothelial growth factor (VEGF) promotes the transdifferentiation of ACs into neurons following a stroke [34]. However, single-cell transcriptomics have recently revealed that the reparative efficacy of ACs is dictated by their functional heterogeneity, which can either promote or impede neural regeneration [35]. Additionally, the inhibitory response of glial cells can dampen the neurogenic function of VEGF.

### 2.4. Roles of PCs in NVU

The unique spatial configuration of PCs within the NVU underscores their pivotal role as gatekeepers of BBB integrity and hemodynamic sensors. PCs maintain extensive direct contact with BMECs, not only governing blood flow and angiogenesis but also actively suppressing vesicular transcytosis through signaling pathways like Mfsd2a to maintain the low-permeability state of the BBB [17]. Kisler demonstrated that PC deficiency triggers chronic hypoperfusion, and longitudinal studies by Montagne further clarified that the loss of PCs precedes neuronal damage by impairing the glymphatic clearance of metabolites (e.g., Amyloid-β), thereby accelerating the progression of AD and PD [36].

Furthermore, although α-SMA expression is predominantly restricted to pre-capillary sphincters, Hartmann confirms that capillary PCs in high-resolution intravital imaging, even those with low or undetectable α-SMA expression (primarily identified by PDGFRβ), still exert fine-tuned control over capillary diameter through calcium-dependent signaling, mediating neurovascular coupling [37].

In essence, PCs are dynamic regulators of the microvascular diameter, and their functional restoration has emerged as a promising therapeutic strategy for mitigating neurovascular dysfunction in NDDs and stroke [36].

### 2.5. The Role of MG in NVU

MG serve as the primary resident immune cells of the CNS, exhibiting dynamic functional profiles that parallel peripheral macrophages while maintaining unique homeostatic signatures [38]. Advances in single-cell transcriptomics from Hartmann have redefined MG activation beyond the simplistic M1/M2 dichotomy, characterizing it instead as a spectrum of state-specific phenotypes, such as disease-associated MG and white matter-associated MG [39]. Beyond immunosurveillance, MG facilitates neurogenesis within the subventricular zone (SVZ) by modulating the niche via the release of trophic factors and chemokines, including BDNF and TGF-β. Current evidence highlights the multifaceted role of MG in neurodegeneration, specifically, they are indispensable for preserving myelin integrity and preventing axonal loss by clearing inhibitory lipid debris [40]. MG serve to shield OGs from sheath-related damage, thereby mitigating nerve degeneration. Conversely, MG can exacerbate neurotoxicity by triggering ferroptosis and chronic neuroinflammation under pathological conditions [41,42]. Furthermore, this chronic neuroinflammatory state often disrupts the integrity of the BBB within the neurovascular unit, triggering the recruitment of circulating peripheral immune cells. By secreting key chemokines, such as C-C motif chemokine ligand 2 (CCL2) and C-X-C motif chemokine ligand 10 (CXCL10), MGs actively attract peripheral monocytes and T lymphocytes into the CNS parenchyma. The subsequent crosstalk between resident MGs and these infiltrating immune cells creates a complex immunometabolic cascade that further exacerbates neurodegeneration [43].

Consequently, the “double-edged sword” nature of MG encompasses both neuroprotective homeostatic support and maladaptive inflammatory responses in the diseased CNS [44].

### 2.6. The Role of OGs in NVU

OGs represent the most abundant cells in white matter. They envelop neuron axons, forming the myelin structure crucial for maintaining the rapid and efficient conduction of electrical signals in neurons. Additionally, OGs enhance the efficiency of neuron excitation and provide essential nutritional support [45,46]. Pathological alterations in OGs can lead to multiple sclerosis in adults. Madhavan and Kamei have enabled the generation of human induced pluripotent stem cell (hiPSC)-derived oligocortical organoids in 3D co-culture, which successfully recapitulate functional myelination and OGs-axon crosstalk within a sophisticated neural niche [47,48].

These models offer powerful platforms for investigating white matter pathologies and remyelinating therapies in a disease-relevant context.

### 2.7. The Role of Neurons in NVU

Within brain tissue, various trophic factors, released by neurons, act upon their own receptors or neighboring neurons, facilitating paracrine signaling [49,50]. The secretion of factors such as platelet-derived growth factor (PDGF), epidermal growth factor (EGF), insulin-like growth factor (IGF), transforming growth factor (TGF), and fibroblast growth factor (FGF) profoundly impacts the nutritional state of the cells surrounding neurons. In physiological scenarios, neurons tightly control and regulate local blood flow through vascular coupling to satisfy the energy and oxygen demands. In a mature brain, neurons play a crucial role in orchestrating the maintenance of the vascular network [51]. Neurons influence local blood flow for various reasons, encompassing growth, interactions with glial cells, physiological activities, and responses to brain injuries.

Chen expanded this understanding by highlighting the role of neuron-derived extracellular vesicles (NDEVs) as a novel mode of communication within the NVU. Unlike traditional soluble factors, these NDEVs encapsulate microRNAs and proteins that can cross the BBB, precisely regulating endothelial TJPs and vascular permeability under stress conditions [52].

Moreover, advanced physiological imaging has revealed that neurons do not solely regulate blood flow via arterioles but also exert fine-tuned control over capillary PCs, a mechanism now considered critical for matching local energy supply with synaptic plasticity during high-frequency neuronal activity [53].

### 2.8. The Role of NSCs in NVU

Historically, the concept of the NVU was primarily centered on mature cellular constituents governing BBB integrity and metabolic coupling. However, emerging physiological evidence necessitates the inclusion of NSCs as an integral, dynamic component of the NVU, particularly within specialized neurogenic microenvironments such as the vascular-NSC niche in the SVZ and granular area of the dentate gyrus within the hippocampus. Within this highly organized niche, NSCs are physically anchored to brain microvessels and are in direct communication with ECs. The resident microvasculature does not merely provide passive oxygen and nutrients, it actively secretes specific angiocrine factors that tightly regulate the quiescence, proliferation, and fate determination of NSCs. In reciprocity, during tissue repair following ischemic injury, activated NSCs engage in extensive paracrine crosstalk with the surrounding vascular and glial components to drive concurrent neurogenesis and angiogenesis. Therefore, incorporating NSCs into in vitro NVU models is critically justified for accurately simulating brain tissue regeneration and evaluating stem-cell-based translational therapies [14,54,55,56,57].

NSCs are a distinct type of stem cell. In the adult brain, they predominantly reside in the SVZ and the granular area of the dentate gyrus within the hippocampus [58,59,60]. NSCs possess the unique ability to self-renew and differentiate into neurons, ACs, and OGs through asymmetric division [61]. Epigenetic regulation directly governs the differentiation of NSCs [62]. Following brain injury, NSCs become activated and differentiated into specific cell phenotypes [63]. However, the inherent NSC population is limited both in quantity and quality, becoming more pronounced with aging and leading to a gradual decline in neurogenesis [60]. Metabolic reprogramming serves as a critical checkpoint for NSCs activation, specifically, the transition from fatty acid oxidation to aerobic glycolysis is essential for the exit from quiescence to the initiation of proliferation [64]. Furthermore, a single-cell RNA sequencing longitudinal study has revealed a previously unrecognized state of “primed” NSCs that exhibit rapid response kinetics following ischemic insult, highlighting a highly heterogeneous plasticity within the niche [65]. In this context, the primed state refers to a transitional phase where NSCs, although phenotypically quiescent, possess a pre-activated molecular signature and metabolic readiness that enables a virtually instantaneous reentry into the cell cycle. To overcome the limitations of the endogenous NSCs pool, bio-orthogonal hydrogels and 3D-printed conductive scaffolds have been developed to mimic the native ECM, significantly enhancing the survival and targeted neuronal differentiation of transplanted NSCs in vivo [66].

Therefore, harnessing the potential for NSCs proliferation and differentiation, in conjunction with various therapeutic approaches, becomes highly advantageous [67,68,69]. In an NVU model, the combination of edaravone and catalpol has been utilized to enhance the overall activity of the NVU and the capacity of NSCs to differentiate into neurons. Illuminating the molecular mechanisms underlying NSC differentiation is essential in the prevention and treatment of ischemic stroke [14].

**Table 1:** Functional comparison of each cell in NVU.

Cell Types	Major Functions	References
**BMECs**	① Constitute blood vessels and BBB. ② Control substance exchange. ③ Secrete angiocrine factors to support neurogenesis. ④ Regulate immune cell trafficking into the brain.	[2,15,16,18,70]
**ACs**	① Form the BBB. ② Drive glymphatic waste clearance via AQP4 channels. ③ Regulate capillary blood flow (neurovascular coupling). ④ Maintain BBB integrity via endfeet signaling.	[71,72,73]
**PCs**	① Form the BBB. ② Control substance exchange. ③ Control the blood flow rate. ④ Regulate capillary diameter (fine-tune local blood flow). ⑤ Modulate immune response and fibrosis after injury. ⑥ Possess stem cell-like properties for tissue repair.	[74,75,76,77,78]
**MG**	① Monitor abnormal life activities of cells. ② Remove harmful substances. ③ Perform continuous immune surveillance (sensing niches). ④ Execute synaptic pruning to shape neural circuits. ⑤ Regulate neuroinflammation and interact with BMECs. ⑥ Clear cellular debris via phagocytosis.	[77,78,79,80]
**OGs**	① Constitute the myelin sheath. ② Improve neurons communication efficiency. ③ Provide metabolic support (lactate shuttle) to axons. ④ Enable adaptive myelination (neural circuit plasticity). ⑤ Modulate neural network synchronization.	[45,46,81]
**Neurons**	① Mediate learning and memory, emotion, consciousness control, and basic life activity control. ② Orchestrate neurovascular coupling (matching blood flow to activity). ③ Regulate BBB integrity via extracellular vesicles (EVs). ④ Release trophic factors.	[49,50,52,70]
**NSCs**	① Proliferate and differentiate into neurons, ACs, and OG. ② Regulate activation via metabolic reprogramming (quiescence-to-active switch). ③ Promote brain tissue recovery.	[61,63]

## 3. Research Progress on In Vitro 3D Models of NVU

### 3.1. General Overview of 3D NVU Models In Vitro

Relying solely on simplified two-dimensional (2D) cell cultures or ethically restricted animal experiments leaves a significant gap in our understanding of the NVU. Standard monolayer cultures simply cannot reproduce the complex spatial architecture and vital cell-to-cell communication required for core functions like BBB integrity and neurovascular coupling. Consequently, the field has shifted toward engineering advanced 3D in vitro systems. By embedding essential NVU cells, such as BMECs, PCs, ACs, and neurons, within environments that replicate the native ECM, these platforms better reflect in vivo physiology. The primary approaches currently include Transwell inserts, 3D matrix gel models, and microfluidic chips. Because each system balances throughput, anatomical accuracy, and dynamic fluid shear stress differently, researchers can select the most appropriate model, whether they are conducting high-throughput screening or investigating the underlying mechanisms of CNS disorders.

#### 3.1.1. Cell Sourcing Strategies for NVU Models

Beyond structural design, the origin of the cellular components is a decisive factor dictating the functional maturation, species relevance, and viable lifespan of in vitro NVU models. Currently, research primarily relies on four main sourcing strategies: primary cells, established cell lines, tumor cells and hiPSC-derived cells. Primary cells, whether of animal or human origin, are highly valued for their immediate display of mature phenotypes, offering robust physiological relevance for short-term functional assays. However, their utility is consistently hindered by limited proliferation capacity, a rapid decline in viability in vitro, and potential ethical or interspecies translational barriers [82].

Conversely, the advent of hiPSC technology has significantly advanced model longevity and the potential for personalized medicine. hiPSC-derived components enable highly scalable, patient-specific disease modeling (such as for AD and PD) and successfully support long-term functional maintenance. Despite these strengths, hiPSC models face distinct methodological challenges, including complex and time-consuming differentiation protocols, inherent epigenetic variability, and a well-documented tendency to exhibit immature or fetal-like transcriptomic phenotypes. A comprehensive comparison of these four predominant strategies is summarized in Table 2, highlighting their specific advantages and inherent limitations to guide optimal model design [83].

#### 3.1.2. General Principles and Methods for Models Validation

Before applying engineered 3D NVU models to downstream disease modeling or pharmacological screening, a comprehensive validation scheme is mandatory to confirm their physiological suitability. The validation principles generally encompass four hierarchical levels: biological integrity, physical integrity, molecular profiling, and functional responsiveness (Table 3).

First, biological integrity ensures the completeness of the cellular composition, confirming the presence, appropriate ratio, and correct spatial arrangement of essential NVU cell types to recapitulate the native microenvironment and provide fundamental neurotrophic effects for cellular survival and network formation. Second, physical integrity focuses on the 3D spatial structure of BBB, which is predominantly evaluated through quantitative TEER measurements and paracellular permeability assays utilizing fluorescent tracers of varying molecular weights (e.g., FITC-dextran) to confirm barrier tightness. Third, molecular profiling requires immunofluorescence or transcriptomic validation to ensure the continuous, polarized expression of critical TJPs (such as ZO-1, Claudin-5, and Occludin) and specific cell-type markers within the 3D matrix. Finally, functional responsiveness is critical to demonstrate physiological relevance. This includes assessing active efflux transport capability via P-glycoprotein (P-gp) substrate assays, evaluating neurovascular coupling by monitoring dynamic changes in vessel diameter or Ca^2+^ signaling, and quantifying the secretion of specific neurotrophic factors following pathological or neural stimuli. Only models that concurrently satisfy these comprehensive benchmarks are deemed suitable for advanced preclinical applications.

#### 3.1.3. Detailed Parameters of the 3D NVU Models In Vitro

To ensure these important details are easily accessible, we have added a brief description in the revised main text above construction and applicability scenarios of 3D NVU models in vitro (Supplementary Table S1).

### 3.2. Transwell Chamber Models

#### 3.2.1. Construction of Transwell Chamber Models

**Figure 2:**
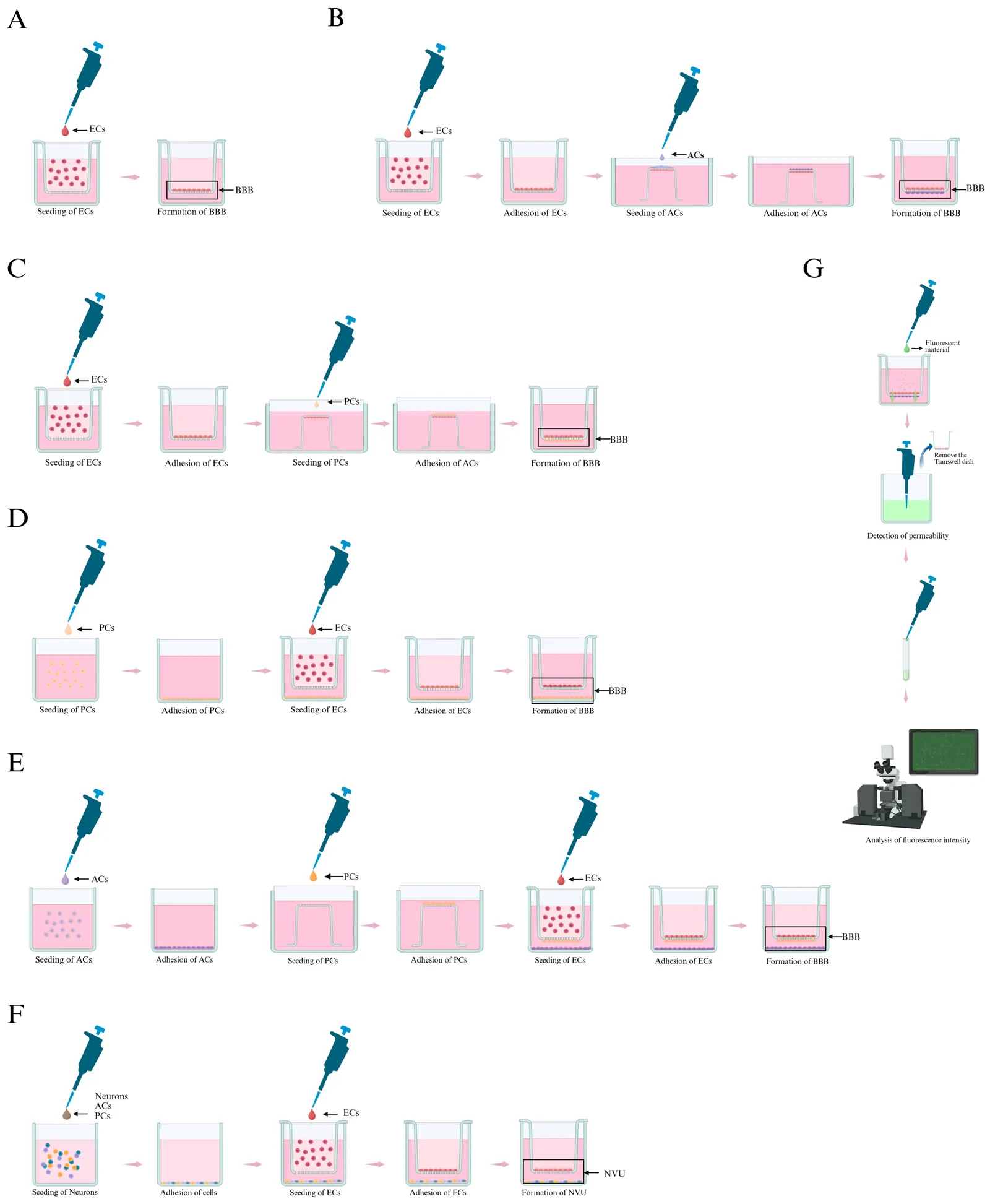
Transwell Chamber NVU models. (**A**) Monoculture Transwell Chamber BBB model. (**B**–**D**) Double-culture Transwell Chamber BBB models. (**E**) Triple-culture Transwell Chamber BBB model. (**F**) Quadruple-culture Transwell NVU Chamber model. (**G**) BBB function test in Transwell NVU Chamber models. Created in BioRender. Gao, Y. (2026) https://BioRender.com/baltv0b.

The Transwell Chamber models are often used to study TJPs expression and BBB permeability functions [84,85,86,87,88,89]. Typically, ECs are seeded onto the inner surface of the Transwell insert. Once the ECs adhere and form a monolayer, other cell types, such as ACs, PCs, or neurons, are seeded on the outer surface of the insert or at the bottom of the well plate. Based on the diversity of co-cultured cells, these models are categorized into monoculture models (Figure 2A), co-culture models (Figure 2B–D), triple-culture models (Figure 2E), and quadruple-culture models (Figure 2F).

#### 3.2.2. Applicability Scenarios

Due to their high-throughput capacity and technical reproducibility, Transwell Chamber models have become indispensable tools in pharmaceutical research for screening CNS drugs based on permeability and for optimizing nanocarrier-based delivery systems [90].

Beyond drug discovery, these platforms are increasingly utilized to simulate pathological states such as AD and PD, ischemic stroke, and glioma, allowing researchers to investigate how barrier dysfunction contributes to disease progression [91]. To achieve this, researchers tailor the Transwell microenvironment by introducing distinct pathophysiological stimuli. Firstly, for modeling ischemia-reperfusion injury (IRI), such as ischemic stroke, researchers frequently employ the OGD/R protocol. By subjecting the models to a transient hypoxic and glucose-free environment (usually 4–8 h), this model effectively reproduces BBB disruption and is heavily used to screen targeted neuroprotective agents. For instance, utilizing an in vitro BBB model constructed by co-culturing ECs and PCs, researchers have evaluated the therapeutic potential of brain microvascular endothelial cell-derived exosomes (EC-Exo). Following OGD/R-induced injury, the administration of EC-Exo significantly restores barrier integrity by increasing TEER and decreasing tracer permeability, while effectively mitigating pericyte dissociation. Mechanistic investigations facilitated by these platforms reveal that such exosomes act as robust neuroprotective agents by activating the Platelet-Derived Growth Factor-Platelet-Derived Growth Factor Receptor Beta (PDGF-PDGFRβ) and Angiopoietin-1/Angiopoietin-2-Tyrosine kinase with immunoglobulin-like and EGF-like domains 2 (AngI/AngII-TieII) signaling pathways, subsequently upregulating essential TJPs and basement membrane proteins. Furthermore, as the research framework evolves, transitioning these OGD/R protocols from standard Transwell systems to advanced matrix gel-based 3D models could provide an even more physiologically accurate NVU microenvironment for screening similar targeted therapeutics [92].

Secondly, for neuroinflammation models, pathological states are typically induced using potent inflammatory mediators such as lipopolysaccharide (LPS). This intervention rapidly compromises barrier integrity and decreases TEER, making it an indispensable tool for investigating the transmigration of peripheral immune cells across the BBB [93]. In practical applications, peripheral blood mononuclear cells (PBMCs) or specific circulating immune subsets (such as monocytes or lymphocytes) can be introduced into the luminal (upper) compartment of the Transwell. Following pathological stimulation, the targeted upregulation of endothelial adhesion molecules captures these circulating cells, allowing researchers to dynamically quantify their transendothelial migration into the abluminal (brain) compartment and to evaluate the efficacy of anti-inflammatory interventions [94].

Lastly, for NDDs models (such as AD and PD), the pathological environment is often established by introducing neurotoxic amyloid-beta (Aβ) oligomers, which allows researchers to longitudinally investigate how barrier dysfunction exacerbates the accumulation of metabolic waste during disease progression. Furthermore, these in vitro platforms are highly instrumental in evaluating transport mechanisms and screening potential therapeutic interventions. For instance, using Transwell BBB models, researchers have demonstrated that post-translationally modified Aβ isoforms (such as iso-Aβ42 and pS8-Aβ42) exhibit enhanced transcytosis across the endothelium compared to intact Aβ. By introducing specific rescue strategies into the microenvironment, such as the RAGE-specific antagonist FPS-ZM1 or endocytosis inhibitors like filipin, researchers can effectively block the receptor-mediated transcytosis of these highly pathogenic peptides. This capability not only elucidates the distinct endocytotic pathways utilized by different Aβ variants but also highlights the potential of these platforms to identify novel pharmacological targets aimed at preventing the peripheral influx of neurotoxic proteins [95].

Furthermore, for CNS infection and neuroinvasion models, researchers aim to elucidate how microbial pathogens breach the brain defenses. In these studies, pathological conditions are typically modeled by inoculating the luminal (vessel) compartment of the Transwell with neurotropic viruses (such as SARS-CoV-2, EV-A71, or neuroinvasive flaviviruses) or bacterial agents. This intervention allows researchers to dynamically track the specific routes of neuroinvasion, whether pathogens cross via direct infection of ECs (transcellular transcytosis), by disrupting TJPs (paracellular leakage), or by hijacking migrating infected immune cells (the “Trojan horse” mechanism) [96].

Consequently, the Transwell system is highly valuable not only for deciphering the fundamental mechanisms of pathogen transport across the BBB [96], but also for screening the permeability and protective efficacy of emerging antiviral therapeutics aimed at preserving barrier integrity [97].

In general, increasing the diversity of co-cultured cell types enhances the structural and functional integrity of the BBB models. The construction of Transwell Chamber models is relatively straightforward, and subsequent analyses, such as fluorescently coupled dextran transmembrane transport assays (Figure 2G) and TEER measurements, are easily performed [84,88,89,98]. However, Transwell Chamber models present distinct limitations: although cell growth occurs in a layered manner, the cells proliferate in a 2D monolayer, so their spatial structure is quite different from that in vivo.

### 3.3. Gel-PDMS-Based NVU Models

**Figure 3:**
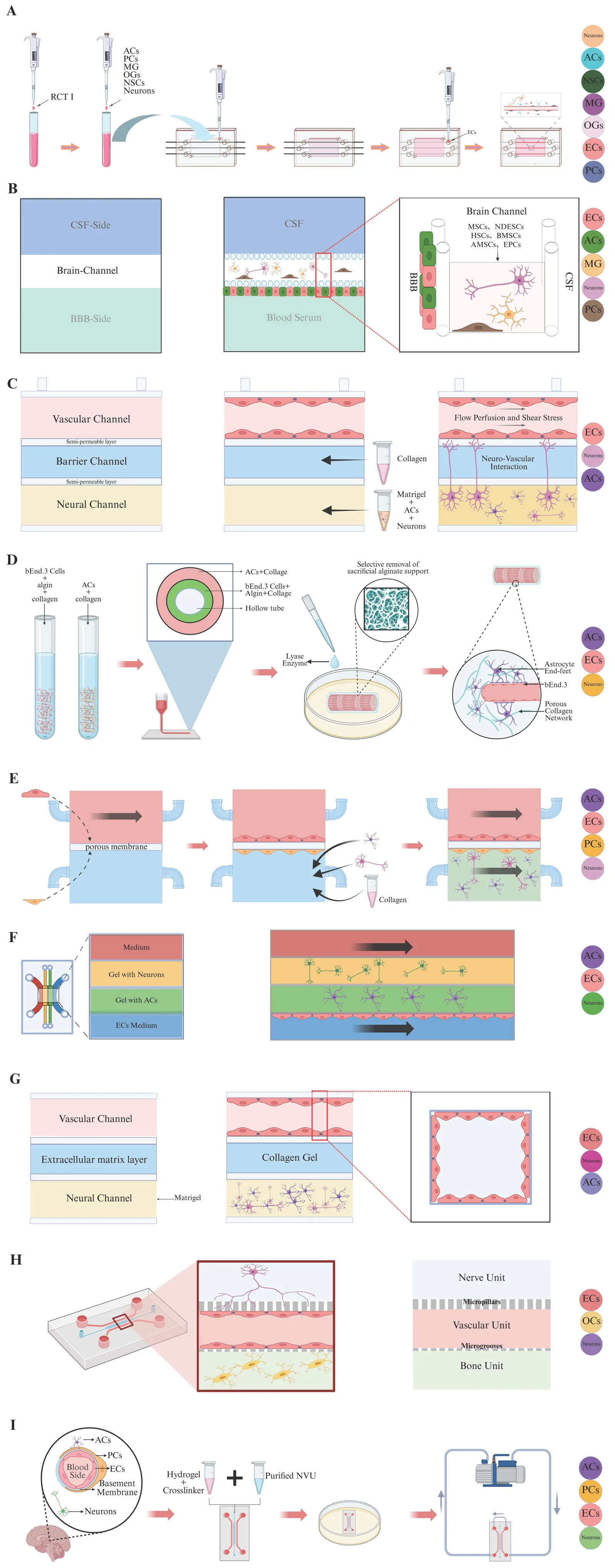
Gel-PDMS-based NVU models. (**A**) The construction process of matrix gel NVU model. (**B**) Cell transplantation nerve vascular structure unit model diagrammatic drawing: A neurovascular-unit-on-a-chip for the evaluation of the restorative potential of stem cell therapies for ischemic stroke. (**C**) Schematic of the BBB self-assembly process featuring a central hydrogel cavity and bilateral perfusion channels. (**D**) Schematic of the construction workflow for the 3D collagen-alginate hydrogel-based NVU model. This experiment incorporated PC12, a type of neurons derived from pheochromocytoma. (**E**) Physical assembly workflow of dual-chamber NVU based on type I collagen hydrogel matrix. (**F**) Schematic of the NVU co-culture model construction based on a four-channel architecture with independent 3D matrices. (**G**) Schematic of spatial distribution and three-dimensional co-culture of NVU within a hydrogel-supported three-channel microfluidic chip. (**H**) Schematic of the physical structure and co-culture of an in vitro Neurovascular Bone (NVB) three-compartment microfluidic chip utilizing a hydrogel matrix. (**I**) Construction and perfusion flowchart of an in vitro human brain microvascular-hydrogel microfluidic chip model. Created in BioRender. Gao, Y. (2026) https://BioRender.com/28foyt0.

Despite the success of static gels in tissue engineering, their lack of fluidic shear stress poses a significant limitation for BBB maturation. To overcome this biophysical deficit, the field evolved towards perfusable gel architectures. The integration of an embedded 3D tubular structure within a rat tail collagen I (RTC I) (Figure 3A) enables the dynamic co-culture of seven distinct immortalized human cell types [55]. PCs, ACs, neurons, MG, OGs, and NSCs were mixed with RTC I, a gel specifically chosen to match the softness and viscoelasticity of native neural tissues, while BMECs were seeded into the cylindrical cavities to line the luminal surface. By optimizing the seeding density of PCs (up to 30% of the total RTC I cell population), this model significantly restricts transendothelial permeability, establishing a highly mature barrier function comparable to physiological conditions. Beyond 3D vascular reconstruction, this perfusable RTC I-based model serves as a highly robust platform for modeling neuroinflammation and investigating multicellular paracrine crosstalk. By delivering pro-inflammatory stimulants, such as LPS, directly through the perfused microchannel, researchers can accurately emulate vasculature-originated neuroinflammation. In this pathological context, the model demonstrated that the presence of the complete NVU cellular niche effectively alleviates LPS-induced BBB disruption, a regulatory protective effect that is entirely absent in BMECs monocultures. Furthermore, integrated cytokine microarray analyses revealed that this multicellular gel environment dynamically regulates immune responses and structural integrity by upregulating specific factors, such as Bruton’s tyrosine kinase and junction-related cytokines. The platform also facilitates the high-resolution morphological tracking of immune cell activation, successfully capturing the branch elongation of MG under inflammatory stress. Consequently, this dynamic NVU model offers unprecedented insights into human-specific physiological mechanisms and provides a powerful tool for high-content drug safety and efficacy screening targeting neuroinflammatory and cerebrovascular diseases.

Building upon the necessity of fluid dynamics, subsequent models recognized that a single perfusable tube is insufficient to capture the multifaceted fluidic interfaces of the human brain, such as the simultaneous circulation of blood and cerebrospinal fluid (CSF). To address this architectural limitation, the development of a dual-liquid NVU chip represents a significant structural advancement. By integrating five key cell types, neurons, ACs, MG, PCs, and ECs, into a compartmentalized design, this innovative platform successfully replicates the complex dual-circulation system of the CNS [56] (Figure 3B). This innovative chip featured three distinct regions: the central brain compartment is separated by two rows of closely arranged microcolumns with attached ECs, while the cerebrospinal fluid (CSF) area and vascular area occupied either side. Both sides facilitated the circulation of solutions, allowing for a more accurate replication of the CNS microenvironment. The dual-liquid NVU model was subjected to the OGD/R condition to simulate IRI. It served as a platform for selecting human stem cells, including neural progenitor cells derived from multipotent stem cells, NSCs derived from embryonic stem cells (NDESCs), hematopoietic stem cells (HSCs), bone marrow-derived mesenchymal stem cells (BMSCs), adipose-derived mesenchymal stem cells (AMSCs), and endothelial progenitor cells (EPCs), for transplantation studies. Examining the inflammatory response, which plays a vital role in post-stroke repair, involved PCR chip analysis. This analysis highlighted the close relationship between inflammatory response genes and post-ischemic stroke repair [99]. Notably, the transplantation of AMSCs increased the expression of inflammation-related genes, while the transplantation of EPCs and BMSCs led to decreased expression. Additionally, HSCs enhanced the expression of CD146 in PCs, which mediated angiogenesis. In summary, the hydrogel-based 3D models improved the accuracy of simulating the pulsatile system and enhanced circulatory function. Moreover, appropriately applied shear forces promoted the expression of TJPs, thereby enhancing BBB function.

Continuing the evolution of compartmentalized chip designs, there is a continuous effort to shield delicate neural cells from unphysiological direct fluid flow. To achieve this, the deployment of a full-3D NVU-on-a-chip model utilizing a 3-lane OrganoPlate provides a highly optimized architecture to reproduce complex neural-vascular interactions [100]. This tri-culture system establishes a biomimetic neurovascular interface by organizing BMECs, ACs, and neurons into a compartmentalized, parallel spatial arrangement. In this method, a unique double-gel architecture was employed: the bottom channel was loaded with Matrigel encapsulating primary human ACs and Lund human mesencephalic-derived neurons, while the middle channel contained a collagen I gel to physically separate the neural compartment from the vascular channel. Primary human BMECs were then seeded in the top channel against the collagen to form a perfusable tube, which was subjected to bidirectional continuous flow via a rocker (Figure 3C) [100]. This model was primarily applied to simulate neuroinflammation-induced BBB disruption and PBMCs extravasation, as well as to serve as a screening platform for evaluating the transcytosis of heparin-binding EGF-like growth factor (HB-EGF)-targeted nanobodies, a class of small, single-domain antibodies. Previous studies have shown that the embedded neural cells formed extensive 3D networks exhibiting spontaneous calcium waves, and the perfusable endothelial tube displayed functional P-gp-mediated efflux. Furthermore, the neural cells successfully projected axons and migrated through the collagen to establish direct contact with the tube. A significant advantage of this full-3D platform lies in its optimized physical structure and microenvironment. It provides a true 3D ECM that shields neural cells from unphysiological direct fluid flow, while simultaneously utilizing this dynamic flow to minimize the ineffective adhesion of functional molecules (e.g., nanobodies) to non-target substances. This dual benefit offers superior physiological relevance compared to traditional Transwell systems [101]. However, the model has distinct drawbacks, including the absence of critical NVU components like PCs and MG, limitations in model longevity due to gel degradation over prolonged culture (collapsing after 21 days), and architectural constraints that make retrieving basal medium samples from the “brain side” difficult for calculating exact permeability values [100].

As microfluidic designs grew in complexity, the reliance on manual gel casting revealed limitations in spatial precision and mechanical stiffness, which often failed to match the ultra-soft nature of the native brain ECM. This prompted a shift towards advanced biofabrication. Consequently, the implementation of 3D coaxial bioprinting, coupled with a dynamic stiffness regulation strategy-provides an unprecedented approach to construct structurally precise NVU models that faithfully mimic native tissue mechanics. This model employed a two-stage biofabrication approach: During the printing stage, a low-viscosity alginate/type I collagen composite bioink was used to construct high-resolution (approximately 10 μm) hollow coaxial structures via embedded 3D printing technology. During the culture phase, alginate lyase was used to selectively degrade the alginate gel, reducing hydrogel stiffness from the printed steady state to approximately 1 kPa, thereby mimicking the extremely soft ECM environment of the brain (Figure 3D) [102,103]. This model was primarily used for studying BBB function and neuropharmaceutical screening [104]. By successfully decoupling printing fidelity from final gel stiffness, this biomanufacturing strategy unlocks several advanced application scenarios. First, it provides an optimal platform for mechanobiological studies, enabling researchers to investigate how an ultra-soft brain-like microenvironment (~1 kPa) directly regulates cellular morphogenesis, such as ACs branching and targeted end-foot projections. Second, the highly reproducible, anatomically defined coaxial architecture makes it an ideal candidate for modeling complex physiological and pathological states of the NVU, allowing for precise investigations into structural barrier dysfunction mechanisms. Finally, because this framework successfully recapitulates both macromolecular-selective permeability and native cell-matrix interactions without relying on artificial porous membranes, it serves as a high-fidelity, scalable in vitro platform for neuropharmaceutical screening and the evaluation of targeted CNS drug delivery systems. Studies have shown that ECs in the model highly expressed TJPs (ZO-1, Occludin, and Claudin-5) and exhibited macromolecular-selective permeability. Concurrently, ACs adopted a branched morphology and established direct physical contact and interactions with ECs [105]. The primary advantage of the model lies in balancing shape precision during bioprinting with cellular growth requirements, eliminating the need for traditional rigid porous membranes. This enabled controlled multicellular spatial distribution and mimics the stiffness of the in vivo microenvironment. However, its limitations include an incomplete barrier function and a passive diffusion rate higher than some literature reports, potentially related to gaps formed by ECs self-organization within the 3D gel. Future improvements should focus on optimizing multi-nozzle printing and multi-component ratios (e.g., incorporating PCs) to enhance the complexity and functionality of the model [106].

While advanced biofabrication resolves structural precision, modified macro-architectures remain essential to facilitate distinct functional assays, such as independent apical and basolateral sampling for metabolic profiling. Addressing this specific analytical need, the dual-chamber NVU organ-on-a-chip framework serves as a robust gel-based 3D culture model. This architecture employs a PDMS device partitioned by a porous (0.2 μm) polycarbonate membrane, enabling precise physical separation and compartmentalized analysis (Figure 3E) [107]. Primary human BMECs were seeded in the bottom vascular compartment, while primary human ACs and PCs were seeded above the membrane. To authentically replicate 3D brain parenchyma, researchers layered a collagen gel containing hiPSC-derived neurons and co-differentiated ACs atop the structure (Figure 3E) [107]. This model primarily investigates barrier responses to neuroinflammation driven by LPS and cytokine mixtures, along with their metabolic consequences. Results revealed a time-dependent barrier response to inflammation: initial inflammatory exposure severely disrupted barrier integrity and TJPs expression. Yet, over time, the barrier demonstrated a unique spontaneous partial recovery capacity. Specifically, by interfacing this microfluidic methodology with advanced analytical tools like ultra-performance liquid chromatography-ion mobility-mass spectrometry (UPLC-IM-MS), the platform excels in real-time metabolomics and circulating biomarker discovery. It enables the dynamic mapping of chamber-specific metabolic shifts, such as the severe depletion of essential neurotransmitter precursors (e.g., tryptophan) and key redox metabolites (e.g., ascorbic acid), under acute pathological stress. This strategy renders the model highly applicable for investigating the metabolic dysfunctions underlying NDDs or traumatic brain injuries. Furthermore, the capacity for independent and continuous sampling provides an ideal analytical scenario for high-resolution pharmacokinetic profiling, allowing for the precise tracking of drug transit, metabolic consumption, and multicellular therapeutic responses across the BBB. Furthermore, integrating this model with advanced mass spectrometry techniques successfully yielded chamber-specific dynamic metabolic pathway profiles. A significant advantage of this model was the dual-chamber continuous perfusion system, enabling independent fluid flow and real-time collection of effluent samples from both the brain and vascular sides.

Alternatively, to eliminate the reliance on artificial porous membranes entirely while maintaining compartmentalization, the deployment of a 3D NVU chip (NVC), functioning as a pure gel-based 3D culture model, authentically recreates the in vivo NVU [108]. The NVC was constructed using a PDMS device featuring four parallel interconnected microchannels. Within this model, primary rat ACs and neurons were encapsulated in separate central 3D collagen hydrogels to simulate brain parenchyma, while human cerebral microvascular endothelial cells (hCMEC/D3) were cultured in adjacent fluid channels to form vascular walls (Figure 3F) [108]. The model was employed to quantitatively assess neurite outgrowth and evaluate the effects of neuroactive compounds (e.g., monosodium glutamate) on neuronal activity following BBB crossing. Specifically, by segregating neural and glial populations into distinct but communicating 3D compartments, this platform is ideally suited for deciphering intercellular paracrine signaling, such as how AC-derived factors dynamically regulate neurite arborization and neural network maturation. Furthermore, the integration of functional readouts like real-time calcium imaging and c-Fos expression profiling establishes this model as a robust platform for neuropharmacological screening. It allows researchers to continuously monitor the neuroprotective or neurotoxic downstream effects of therapeutics or toxins precisely after they permeate the BBB. Additionally, the modular gel design provides a foundational framework for personalized medicine. It supports the future integration of patient-derived hiPSCs to reconstruct disease-specific neurovascular niches for evaluating targeted drug efficacy. Previous studies have shown that hCMEC/D3 formed a size-selective permeable barrier, while 3D-cultured neurons exhibited functional calcium oscillations and extensive neurite branching. A significant advantage of this model is the multi-channel design, enabling independent manipulation of specific cell types and simultaneous assessment of barrier permeability and downstream neural function. However, drawbacks include reliance on cross-species co-culture (rat neurons with human ECs, which may adversely affect physiological relevance). Additionally, the system lacks flow-mediated shear stress and TEER measurements to further validate barrier integrity.

Representing a highly integrated approach to hydrogel-supported NVU modeling, a tri-culture model termed NVU on-a-chip utilizes the OrganoPlate three-channel platform (Figure 3G) [109]. By incorporating a central ECM gel lane to physically separate and support the vascular and neural channels, this model bridges the gap between basic compartmentalization and high-throughput platformization, effectively resolving the engineering bottlenecks of complex external pumps. This pump-free, microtiter plate-based architecture significantly expands its application scenarios, particularly in modeling acute neurovascular pathologies and executing large-scale drug screening. First, for in vitro disease modeling of acute insults like ischemic stroke, the platform excels at recapitulating the core biochemical features of the ischemic cascade, including oxidative stress, energy failure, and subsequent barrier breakdown. To evaluate the applicability of the model in disease research, a triple intervention approach combining chemically induced hypoxia (via antimycin A), a sugar-free medium (low glucose), and cessation of fluid perfusion was employed to simulate ischemic stroke [110,111]. Studies have shown that severe disruption of BBB function under simulated stroke conditions (35-fold increase in apparent permeability), a 7.3-fold decrease in mitochondrial membrane potential, and significant reductions in adenosine triphosphate (ATP) levels were observed on both the blood and brain sides. Second, the system demonstrates immense potential in high-throughput phenotypic screening for neuroprotective and BBB-stabilizing therapeutics. Because it is fully compatible with high-content live-cell imaging, investigators can dynamically monitor the restoration of mitochondrial membrane potential and real-time changes in endothelial permeability. This methodology enables the rapid efficacy evaluation of candidate compounds (e.g., free radical scavengers) against IRI. Furthermore, heavily relying on the structural properties of its central hydrogel matrix, this strategic setup provides an ideal 3D environment for investigating immune cell transendothelial migration and dynamic neuroinflammatory responses. The primary advantage of this model lies in its pump-free, gravity-driven bidirectional perfusion system. A single-type cell culture plate accommodates 40 chips for parallel operation, making this relatively high-throughput design well-suited for routine experiments and automated drug screening. However, the limitations of the model include bidirectional flow characteristics, which do not match the unidirectional physiological state of cerebral blood flow in vivo. Furthermore, compared to traditional pump-and-syringe systems, NVU on-a-chip model lacks complete precise control over blood flow parameters such as fluid shear stress.

Expanding upon 3D ECM applications, the research focus naturally expanded from isolated NVU simulations to multi-organ-on-a-chip frameworks that capture systemic physiological interactions. Reflecting this broader perspective, a microfluidic NVU model platform named the NVB chip was developed to simulate the NVU within an inflammatory bone microenvironment. This compartmentalized strategy is specifically engineered to investigate the complex biochemical crosstalk between the sensory nervous system and the bone niche under pathological conditions, particularly mimicking disease models such as rheumatoid arthritis or bone metastasis. This model comprises three interconnected channels: the neural zone contains mouse embryonic dorsal root ganglion (eDRG) explants, with axons physically separated from cell bodies via microgrooves; the vascular zone strategically employs a 3D collagen/fibrin hydrogel to embed human umbilical vein endothelial cells (HUVECs), which is restrained by microcolumn structures to support functional lumen formation; and the bone zone cultured mature mouse primary osteoclasts on collagen-coated surfaces (Figure 3H) [112]. To establish a physiologically relevant neuroinflammation-bone disease model, the osteoclast compartment is stimulated with pro-inflammatory cytokines such as IL-1β, effectively triggering a pathological cascade that replicates the microenvironment of chronic bone inflammation. Previous studies have shown that inflamed osteoclasts not only increased BBB permeability but also significantly promoted sensory nerve axonal sprouting and extension through the hydrogel matrix toward the vascular zone, a critical morphological marker associated with the development of inflammatory bone pain. Furthermore, the NVB chip serves as a sophisticated analytical platform for deciphering the mechanisms of peripheral pain perception and vascular hyperpermeability that are otherwise difficult to isolate in systemic animal models. The study further validated drug screening in the model by delivering ibuprofen-loaded poly (lactic-co-glycolic acid) nanoparticles, successfully reversing inflammation-induced abnormal axonal growth. Consequently, this platform provides a robust methodology for evaluating the efficacy of targeted nanomedicine delivery systems and identifying novel therapeutic interventions aimed at alleviating bone-associated pain and structural degradation. The primary advantage of this model lies in its pioneering integration of fully differentiated primary osteoclasts into a hydrogel-supported NVU model containing both blood vessels and sensory nerves, providing a highly complex in vitro platform for studying bone inflammation, pain perception mechanisms, and nanomedicine delivery [113,114]. However, a notable limitation is its reliance on interspecies cell co-culture (mouse and human cells), which has not yet achieved a fully same-typed matching. This may impose constraints on testing certain drugs with high species specificity.

While the aforementioned bottom-up engineering approaches have significantly advanced, completely recapitulating the intricate, concentric cellular layers of native human microvessels remains an ultimate challenge. Representing a paradigm shift towards top-down integration, recent breakthroughs have utilized intact NVU microvessels extracted directly from postmortem human cortical tissue. A study successfully engineered an in vitro human brain vasculature-on-a-chip model to replicate complex NVU structures. Intact NVU as a whole was extracted from cryopreserved postmortem human cortical tissue using a novel enzyme-free filtration method to preserve native structural integrity. These enriched microvessels were then embedded in a peptide-functionalized hydrogel within a custom microfluidic NVU model device. To support vascular growth and mimic physiological hemodynamics, the culture was maintained under recirculating, pulsatile perfusion that simulated endogenous arterial pressure waves, including a characteristic dicrotic notch, for 14 days (Figure 3I) [115]. This approach offered significant insights for cerebrovascular disease modeling and drug screening by faithfully replicating interconnected arteriole and capillary networks within the same matrix. Specifically, because the model directly utilizes cryopreserved tissue, it inherently preserves the distinct pathophysiological signatures of the donors, making it an exceptionally authentic platform to investigate chronic neurodegenerative conditions such as AD, PD, and cerebral amyloid angiopathy (CAA). Additionally, by adjusting the pulsatile perfusion system to simulate abnormal blood flow, this platform can be utilized to investigate acute vascular injuries like ischemic stroke. Distinct from prior self-assembled single-cell models that struggle to form true anatomical layers, this platform demonstrated concentric rings of smooth muscle cells, PC coverage, and AC endfeet, closely matching native in vivo conditions [116]. Furthermore, the model exhibited robust BBB function, with dextran permeability assays showing highly restricted diffusion equivalent to physiological states. This robust barrier integrity positions the model as an optimal in vitro tool for evaluating the transcytosis of large-molecule neurotherapeutics and targeted nanocarrier delivery systems. Beyond these applications, the biomimetic gel framework provides a permissive niche that can be co-cultured with hiPSC-derived neural progeny to evaluate the integration and efficacy of stem cell transplantation therapies. While the model excels at preserving native cell-matrix interactions and 3D architectures, its primary limitations include a reliance on the availability of postmortem donor tissue and the need for future validation regarding active transport mechanisms and distinct molecular signatures. Thus, this model serves as a highly representative and valuable tool for advancing neurovascular research [115,117].

### 3.4. Self-Assembled NVU Models

Gel-based NVU models are typically cultivated in well-plates, polydimethylsiloxane (PDMS) chips, or Transwell chambers to achieve various experimental objectives. In Transwell setups, gel is often mixed with ECs, NSCs, or other NVU cells before being introduced into the system [14]. Specifically, the cell-laden gel is carefully deposited onto the semipermeable membrane of the Transwell insert. The mixture is then incubated at 37 °C for approximately 30 min to trigger thermal polymerization, forming a stable 3D scaffold. Within the gel, ECs interconnect to form vascular-like networks, while NSCs differentiate into neurons, ACs, and OGs [118]. The construct is subsequently maintained in a specialized co-culture medium for 7 to 14 days to allow for structural maturation and cellular interaction. Through self-organization, these cells collectively establish an NVU.

**Figure 4:**
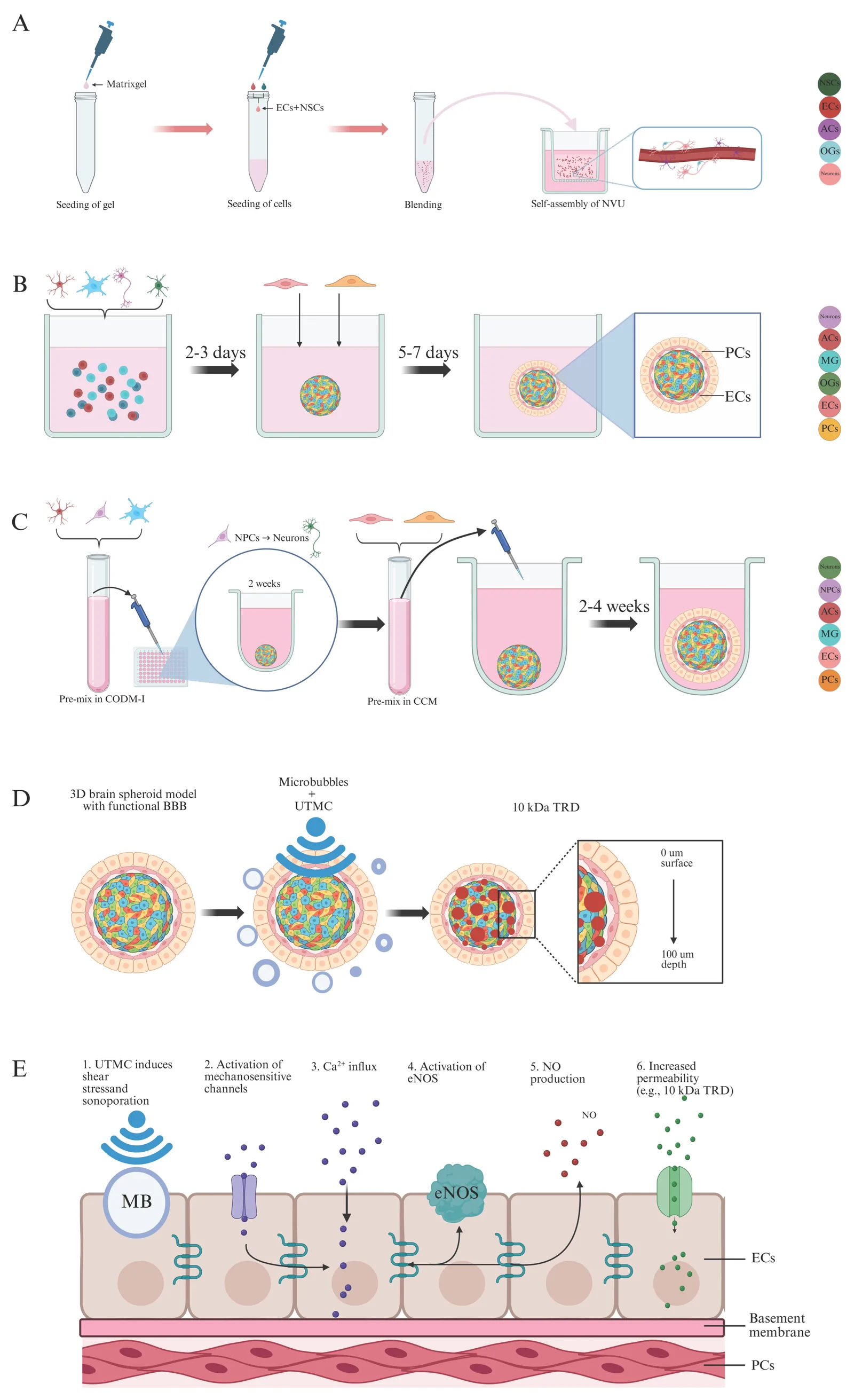
Self-assembled NVU models. (**A**) General view of the construction of tissue transplanted NVU models with primary brain microvascular ECs and NSCs in rats. (**B**) Construction of the six-cell NVU spheroid model. Neurons, ACs, MG, and OGs are first assembled into a brain spheroid core, followed by the addition of ECs and PCs to form a functional BBB-like structure. (**C**) Construction of the developmentally oriented five-cell NVU spheroid model. hiPSC-derived NPCs, ACs, and MG are first co-cultured to generate the brain core through neuronal differentiation, followed by the addition of ECs and PCs to establish a mature BBB-like spheroid. (**D**) Application of the five-cell NVU spheroid model for evaluating ultrasound-targeted microbubble cavitation (UTMC)-mediated drug delivery. UTMC transiently increases BBB permeability, allowing 10 kDa Texas Red dextran (TRD) to penetrate approximately 100 μm into the spheroid. (**E**) Application of the five-cell NVU spheroid model for investigating the mechanism of UTMC-induced BBB opening. Microbubble cavitation activates mechanosensitive ion channels, triggering Ca^2+^ influx, eNOS activation, NO production, and a transient increase in BBB permeability. Created in BioRender. Gao, Y. (2026) https://BioRender.com/uckamc6.

The initial application of matrix gel-based NVU models focused on providing a biomimetic 3D microenvironment that leverages the self-organization of primary cells. For instance, a pioneering 3D gel-based NVU framework was developed by co-encapsulating primary rat NSCs and BMECs at a defined density ratio. This model spontaneously assembled into integrated networks of CD31^+^ vascular-like structures and differentiated neural components, recreating the complex physical interactions and BBB characteristics observed in vivo. Crucially, this gel-based model serves as a highly effective platform for multifactorial pathological modeling and pharmacological screening. By subjecting the construct to OGD/R within an anaerobic chamber using deoxygenated, glucose-free medium, researchers can accurately simulate the pathological cascade of ischemic stroke. Using this OGD/R model, the platform was successfully applied to screen neuroprotective therapeutics, demonstrating that VEGF could restore BBB integrity and promote neuronal axon elongation, while the free radical scavenger edaravone effectively mitigated oxidative stress by suppressing ROS production and apoptosis. Furthermore, extending beyond in vitro mechanistic studies of paracrine crosstalk (e.g., the mutual upregulation of glial cell line-derived neurotrophic factor (GDNF), and angiopoietin-1 (Ang-1)), this model has shown profound potential in tissue engineering and regenerative medicine. When utilized as an active graft transplanted into the infarct cavity of Middle Cerebral Artery Occlusion rat models, the 3D gel-based NVU survived in vivo, significantly reduced infarct volume, improved neurological deficit scores, and actively stimulated host angiogenesis and neurogenesis. These findings underscore the optimal applicability of matrix gel models not only in elucidating neurovascular pathology but also as a translational vehicle for CNS disease transplantation therapies (Figure 4A) [14].

To more accurately replicate the microenvironment of the adult cerebral cortex in vitro, the development of complete NVUs containing a variety of supporting cells has become the focus of research on 3D spheroid models, which can be considered a special series of gel-based NVU models. In 2018, Nzou et al. first reported a 3D spheroid model containing all six major cell types of the cerebral cortex (ECs, PCs, ACs, MG, OGs, and neurons). The model was constructed using an innovative “staged assembly” strategy to address the issue of incomplete ECs coverage resulting from simple co-culture, thereby ensuring the integrity of the outer barrier. First, human astrocytes (HA), human microglia (HM), human oligodendrocytes (HOs), and human neurons (HN) were mixed in a hanging-drop culture plate and co-cultured for 48 h, allowing them to self-assemble into a substantial neuroglial sphere core. Subsequently, in the second stage, human brain microvascular endothelial cells (HBMECs) and human brain microvascular pericytes (HBVPs) are precisely introduced to tightly envelop the periphery of the preformed neural core (Figure 4B) [119].

This staged assembly mechanism promotes the specific colonization of ECs and PCs on the surface of the spheroid, forming a tight barrier coating. This peripheral barrier not only stably expresses TJPs such as ZO-1 and Claudin-5, as well as adherens junction proteins such as VE-cadherin, but also possesses key transporters such as P-gp and glucose transporter 1 (GLUT1), highly replicating the physical barrier and selective transport characteristics of the in vivo BBB [119].

In terms of applications, this six-cell NVU platform has demonstrated tremendous potential in modeling complex diseases and high-throughput neurotoxicity screening. First, in the modeling of ischemic brain injury, this model vividly reveals the physiological degeneration of the barrier under pathological conditions. Studies have shown that after 24 h of culture under hypoxic conditions (0.1% oxygen), the spatial distribution of TJPs and adherens junction proteins (such as ZO-1, Claudin-5, β-catenin, and VE-cadherin) on the surface of the spheroids was significantly disrupted, successfully simulating the barrier leakage and damage caused by clinical ischemia [120].

Furthermore, this platform was used to rigorously evaluate the charge selectivity of the BBB and neurotoxicity defense capabilities. In inorganic mercury toxicity tests, spheroids with an intact BBB effectively blocked the entry of hydrophilic, positively charged mercury ions, thereby protecting deep-layer neurons from ATP depletion and cell death. Furthermore, the platform was successfully applied to a neurotoxicity model of PD: the lipophilic prodrug MPTP was able to readily cross the BBB in this model, where it was subsequently metabolized into the neurotoxic MPP^+^, leading to significant cell death within the spheroids [121].

In contrast, when hydrophilic MPP^+^ was added directly to the culture medium, cells inside the spheroids did not exhibit significant toxic responses due to the charge repulsion and physical barrier provided by the intact BBB. These applications fully demonstrate the exceptional physiological relevance of this multicellular 3D model in evaluating the permeability and toxicological effects of drugs targeting the CNS.

With technological advancements, the application of this model has been further expanded to the highly complex fields of cutting-edge nanodelivery and toxicological assessment. Sokolova et al. directly adopted this highly biomimetic 3D multicellular platform to systematically investigate the transport mechanisms of novel ultrafine gold nanoparticles (metal core diameter approximately 2 nm, hydrodynamic diameter 3–4 nm) across the BBB. The study found that a fully functional peripheral ECs/PCs barrier strictly blocks the infiltration of free small-molecule fluorescent dyes (FAM-alkyne), yet these ultrafine gold nanoparticles effectively overcome the barrier and, driven by a concentration gradient, rapidly diffuse into and accumulate in the deep neural parenchyma core of the sphere. This dynamic transport characteristic not only validates the physical feasibility of ultrafine nanomaterials crossing the BBB but also establishes the core evaluative value of this six-cell sphere model in screening innovative drug delivery systems for the CNS [122].

Furthermore, this extended study organically integrated targeted drug delivery with a pathological injury model, providing an in-depth exploration of pharmacokinetic evolution under ischemic conditions. After 24 h of culture under severe hypoxia (0.1% oxygen) simulating ischemic stroke, the model vividly reproduced the damage and breakdown of the BBB: under pathological conditions, not only did the trans-barrier cellular uptake of gold nanoparticles surge, but even free dye molecules, which are completely blocked under normoxic conditions, exhibited significant parenchymal leakage. Furthermore, in conjunction with ATP metabolic activity assays, this model simultaneously confirmed that gold nanoparticles did not induce significant cytotoxicity in the complex network of brain cells during the highly efficient permeation process, demonstrating ideal biosafety.

However, although this “stepwise rapid assembly” strategy has greatly improved cellular complexity and formation efficiency, the direct assembly of pre-differentiated cells has, to some extent, compromised the ability of neurons to undergo in situ development, naturally differentiate, and establish physiological interaction networks in three-dimensional space. Furthermore, with the advancement of brain drug delivery technologies, research on the regulation of BBB permeability urgently needs to expand beyond traditional chemical stimulation to include non-invasive physical interventions, and to thoroughly elucidate the transient molecular cascade reactions involved.

In line with this trend, Paranjape et al. proposed a highly physiologically relevant “developmentally oriented” five-cell hybrid sphere NVU model in 2024 and successfully applied it to the mechanistic study of ultrasound-targeted microbubble cavitation (UTMC), a cutting-edge physical permeation enhancement technology. Unlike the rapid neuronal assembly strategy employed by Nzou et al., this model pre-mixes immature hiPSC-derived neural progenitor cells (NPCs) with ACs and primary MG, allowing for 3D in situ neuronal differentiation and network development within the spheroid for up to two weeks. ECs and PCs are then introduced to envelop the spheroids for an additional two weeks, ultimately resulting in NVU spheroids with a complete BBB-like structure and functional internal brain parenchyma (Figure 4C) [57].

This 3D platform, featuring a complete and functional BBB, serves as an ideal in vitro screening tool for developing and evaluating new strategies for brain drug delivery. For example, this platform has been specifically used to investigate the feasibility and potential mechanisms of UTMC-induced high permeability of the barrier. Experiments demonstrate that under UTMC treatment, macromolecular model drugs (such as 10 kDa TRD) can effectively penetrate the BBB and penetrate deep into the interior of the spheroid up to 100 μm, without causing significant damage to cell viability (Figure 4D). Second, as an advanced analytical tool, this model has successfully elucidated the underlying signaling cascades regulating barrier opening: it confirmed that UTMC-induced high permeability is highly dependent on mechanosensitive channel-mediated (Ca^2+^ influx, followed by the activation of the endothelial nitric oxide synthase (eNOS) pathway (Figure 4E). This not only highlights the advantages of drug permeability testing but also demonstrates its immense value in studying biological effects in deep spaces far from the endothelial barrier. Combined with patient-specific iPSC technology, this spheroid model is viewed as a powerful tool for simulating complex neurological diseases such as AD and conducting precision drug testing in the future.

### 3.5. Microfluidic NVU Models

Microfluidic NVU models, often referred to as organ-on-a-PDMS-chip systems without ECM components, primarily rely on PDMS due to its lipophilic, hydrophobic, and non-toxic properties, as well as its permeability to CO_2_ and O_2_, which prevents liquid leakage. Cells were seeded into the inner surfaces of chambers within PDMS chips, and external mechanical pumps were often employed to drive medium circulation, simulating systemic blood flow.

Beyond technical flexibility, these microfluidic NVU model platforms are widely utilized in high-throughput screening for CNS-targeted drugs and the evaluation of nanoparticle-mediated delivery systems [90]. Furthermore, they serve as sophisticated tools for modeling neurovascular pathologies, such as AD and PD, ischemic stroke, and neuroinflammation, by accurately simulating shear-stress-induced barrier maturation under biomimetic flow [123].

**Figure 5:**
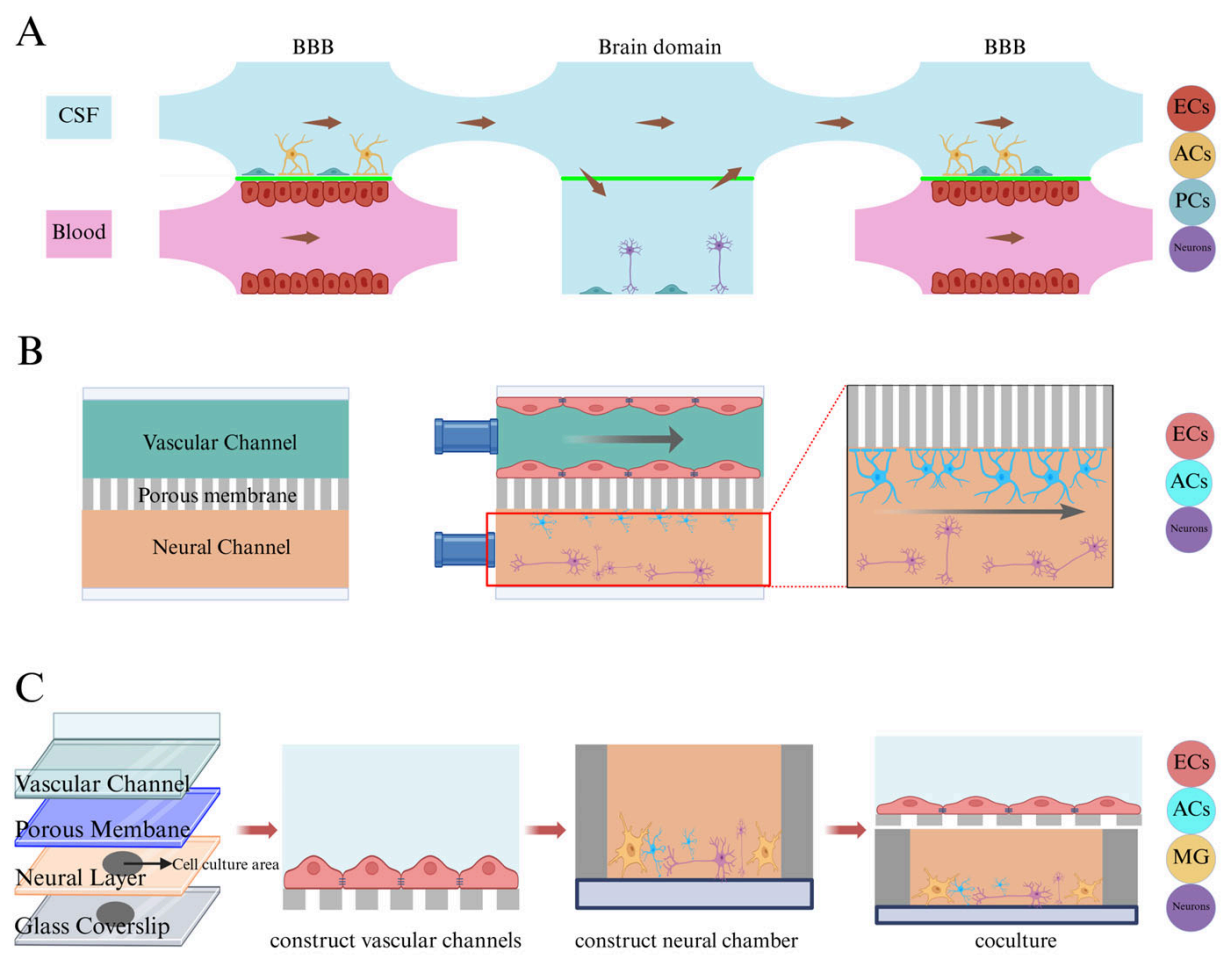
Microfluidic NVU models. (**A**) The construction process of BBB-brain-BBB structure NVU model. (**B**) Schematic diagram of the multicellular 3D NVU-on-a-chip model. (**C**) Schematic of the construction and co-culture workflow of the modular NVU-on-a-chip. Created in BioRender. Gao, Y. (2026) https://BioRender.com/jao8oh5.

As the foundational architecture for microfluidic modeling, early planar multi-compartment designs established the principle of precise spatial segregation. For instance, cells were cultured within a classic coupled microfluidic NVU, incorporating ECs, ACs, PCs, and neurons (Figure 5A) [13]. The model was divided into three zones: the vascular zone, perivascular zone, and nerve zone. The vascular zone was modeled by a single layer of ECs, while the perivascular zone was represented by ACs and PCs, separated from ECs by a semipermeable membrane. Methamphetamine (Meth, an addictive drug) was employed to induce damage to both the BBB and nerve function at various concentrations. In line with prior research, low Meth doses caused reversible BBB damage [124,125]. Specifically, the linked configuration demonstrated that Meth selectively disrupted the influx transport mechanisms of the BBB, enabling the transmigration of macromolecules (such as anti-GluR2 antibodies) into the neuronal compartment, while the efflux transport mechanisms of the BBB remained structurally intact. This highlights its utility as a screening platform for localized drug target effects and region-specific barrier vulnerabilities. Second, the platform serves as an advanced analytical tool for investigating multicellular metabolic coupling. By integrating ^13^C-isotope tracing and untargeted mass spectrometry, the multi-chip strategy successfully elucidated that vascular and perivascular cells secrete metabolic intermediates (such as lactate and pyruvate) that directly regulate and enhance the neuronal synthesis of crucial neurotransmitters, including GABA, glutamine, and glutamate. This capability makes the system highly advantageous for modeling neurometabolic alterations in complex pathologies like neurodegeneration and stroke. This model exhibited excellent barrier and circulatory functions, and the semipermeable membrane facilitated separate testing of cells and metabolites in each domain. Subsequent stem cell engineering breakthroughs have enabled the transition from primary cells to hiPSC-derived NVU components. Modern chips now integrate micro-electrodes for continuous, real-time monitoring of TEER, providing a highly dynamic assessment of barrier integrity and metabolic coupling that was not possible in earlier static-like models [83].

In parallel with structural and systemic expansions, another critical evolutionary branch has focused on overcoming the limitations of traditional endpoint assays by integrating advanced biosensors. For example, a specialized NVU-on-a-chip model was designed specifically for the in situ detection of neuroinflammation. This model consists of two parallel microchannels separated by a permeable porous membrane. The upper channel is lined with hBMECs to mimic vascular structures, while the lower channel is seeded with human ACs and neurons differentiated from hiPSCs-derived neural progenitor cells (hiPSCs-NPCs), representing the neural tissue compartment (Figure 5B) [126]. The study induced inflammatory responses within the microchannels using LPS. This specific intervention strategy serves as an effective model for evaluating the acute neuroinflammatory cascades implicated in severe systemic infections breaching the BBB, as well as the chronic neuroinflammation characteristic of neurodegenerative disorders like AD and PD. One study has shown that LPS intervention caused disruption of TJPs, a significant decrease in TEER, increased BBB permeability, and a substantial decline in neuronal survival. A key advantage of this model lies in its innovative underlying sensor module design. By integrating reduced LPS with fluorescently labeled graphene oxide aptamers, it enabled non-destructive (without sacrificing cells) real-time, highly sensitive quantitative detection of pro-inflammatory cytokines (specifically multiplexing the continuous detection of TNF-α, IL-6, and IFN-γ via Förster resonance energy transfer) secreted by ACs and neurons. This real-time detection strategy fundamentally transforms pharmacological evaluation by allowing researchers to dynamically track the temporal kinetics of cytokine release alongside barrier dysfunction and neuronal death within the exact same sample over time. Furthermore, this platform has been explicitly validated for therapeutic screening, for instance, the administration of the anti-inflammatory drug minocycline successfully attenuated cytokine secretion and rescued neuronal viability, demonstrating its robust applicability for evaluating targeted immunomodulatory neuro-therapeutics. This rendered the model more accurate than traditional animal models in reflecting the human neurovascular microenvironment [127].

Finally, seeking to balance structural complexity with operational flexibility and low sample consumption, alternative architectures have emerged based on vertical modularity. A prominent example is a modular NVU-on-a-chip microfluidic model that employs a vertically stacked architecture, comprising a parenchyma chamber made of PDMS and a vascular channel separated by a microporous polycarbonate membrane (Figure 5C) [128]. This “plug-and-play” modular strategy is particularly advantageous for dissecting complex, multi-step pathological cascades, establishing the platform as a robust in vitro surrogate for studying drug-induced neurotoxicity, traumatic brain injury, and NDDs driven by systemic immune responses. This model was employed to study and simulate neuroinflammatory responses. Previous studies have shown that systemic injection of the pro-inflammatory factor TNF-α through the vascular layer for 6 h induces high permeability in the BBB and significantly activates approximately 75% of resident MG and ACs on the neural side, successfully reproducing a tissue-mimetic response resembling neuroinflammation. Crucially, by allowing independent access to both the vascular and parenchymal compartments, this structural strategy enables researchers to longitudinally track the secondary neurotoxic effects resulting from activated glia, providing a highly targeted framework for screening neurotherapeutics designed to interrupt blood-brain pathological crosstalk. The advantages of the model include a modular semi-open design enabling easy seeding of diverse cell types and adjustment of cell ratios (similar to standard 96-well plate formats). It also offers extremely low sample consumption (50~100 μL), compatibility with high-resolution microscopic imaging, and the ability to permit dynamic fluid perfusion (e.g., inflammatory stimuli), features absent in traditional Transwell chamber models. However, the co-culture system lacks PCs and physiological shear stress stimulation. The manual assembly process is relatively susceptible to contamination, and introducing high shear stress requires additional robust clamping due to the absence of a tight seal [56,101,129].

A comprehensive comparison of the specific applicability, advantages, and inherent limitations among these four main types of in vitro 3D NVU models is systematically summarized in Table 4.

## 4. Conclusions and Future Perspectives

### 4.1. Current Challenges and Technical Limitations

While current 3D in vitro platforms successfully replicate much of the spatial architecture for NVU, they still face practical hurdles. Crucially, a significant technical limitation in many existing platforms is the inadequate maturation of barrier function. When an in vitro model lacks a sufficiently tight physical barrier, often indicated by abnormally low TEER values or discontinuous expression of TJPs, it artificially creates a “leaky” phenotype.

Utilizing these compromised models for screening CNS-targeted drug delivery systems or investigating pathogen neuroinvasion can lead to severe false-positive results, causing researchers to substantially overestimate the actual transcytosis capacity of therapeutic agents or the true neuroinvasiveness of microbial pathogens.

Furthermore, there is a major issue regarding the reproducibility and the inherent variability of these complex systems. The absence of standardized cell sourcing and scaffold protocols makes it difficult to reproduce or compare data across different laboratories. For instance, the use of undefined biological scaffolds, particularly animal-derived hydrogels like Matrigel or collagen, introduces significant batch-to-batch variability and can non-specifically bind drugs, thereby skewing pharmacokinetic data.

Beyond matrix-related inconsistencies, a prevalent yet frequently under-discussed field-wide limitation is the reliance on cross-species co-culture systems. In an effort to rapidly assemble complex structures, many advanced platforms construct the neurovascular unit by integrating animal-derived neural cells (such as rodent ACs or neurons) with HBMECs.

While this strategy bypasses the challenges of sourcing specific primary human tissues, the inherent interspecies molecular mismatch at the neurovascular interface severely compromises physiological relevance. This biological discrepancy introduces significant confounding variables, particularly when screening species-specific neurotherapeutics or evaluating targeted delivery systems, ultimately hindering the translational value of these models from bench to clinic.

To overcome these systemic bottlenecks, practical standardization efforts across the field are actively evolving. Rather than merely conceptualizing uniformity, current initiatives are actively shifting toward replacing highly variable animal-derived matrices with fully defined synthetic or recombinant hydrogels to eradicate batch-to-batch inconsistencies. Concurrently, ongoing efforts are establishing universal consensus protocols for cellular differentiation to ensure phenotypic stability, alongside promoting microfluidic architectures with standardized, commercially adaptable dimensions. These targeted, practical standardizations are essential to enable reliable cross-laboratory data validation and to successfully transition these platforms into industrial-scale, high-throughput screening environments.

### 4.2. Summary and Future Outlook

The multicellular-component NVU in vitro model has found valuable applications in the field of CNS disease research. It contributes to the study of neuroinflammation, BBB permeability, absorption of psychotropic drugs, metabolic processes, and the potential of stem cell transplantation for the treatment of NDDs and ischemic stroke. Using 3D in vitro NVU models, researchers can examine and simulate physiological processes and pathologies, observe morphological changes, assess tissue function, and delve into the underlying molecular mechanisms. These 3D in vitro NVU models are important tools for clinical drug screening, stem cell transplantation, and organ transplantation studies, all of which have important research implications (Table 2). This article focuses on the basic characteristics, construction methods, and application scenarios of several main types of 3D NVU models in vitro. The NVU model grown in gel or other media can simulate the real internal environment to a high degree, and the 3D structure tends to be realistic, which provides new methods and experimental approaches for the physiological and pathophysiological research of the CNS.

Looking forward, to elevate the translational novelty of NVU models, several original concepts and hypotheses warrant active exploration in future research. A primary frontier lies in the “Asynchronous aging” Hypothesis. Current models predominantly utilize cells of uniform developmental stages. However, we hypothesize that by intentionally co-culturing aged HBMECs with young neural progenitors (or vice versa), researchers could isolate the specific vascular versus neural drivers of age-related neurodegeneration, potentially uncovering novel vascular-rejuvenation targets. Beyond localized cellular aging, the field must expand toward systemic integration, specifically conceptualizing a “Brain-microbiome-immune axis” on-a-chip.

Future iterations should evolve beyond isolated NVU constructs to multi-organ coupled systems. By actively introducing gut-microbiome-derived metabolites (such as short-chain fatty acids) into the vascular flow, researchers can investigate how systemic metabolic shifts distally regulate BBB permeability and microglial polarization. Ultimately, the convergence of bioengineering and computational science offers the prospect of artificial intelligence-driven predictive NVU models. By coupling real-time microfluidic biosensors with machine learning algorithms, future platforms could dynamically map temporal-spatial multi-omics changes, transitioning in vitro NVU models from passive observational tools into predictive engines for evaluating complex CNS drug neurotoxicity.

In summary, this paper has reviewed several common in vitro 3D NVU models, which have shown increasing research significance and provide a good carrier for the research of brain function and disease.

## Figures and Tables

**Table 2:** Comparison of cell sourcing strategies in 3D NVU models.

Cell Source Strategy	Representative Cell	Advantages in 3D NVU Models	Limitations in Function and Maintenance
**Primary cells**	hBMVEC HBVP BMEC Osteoclast eDRG	① Exhibit mature phenotypes immediately. ② Provide high physiological relevance and functional validation in short-term cultures.	① Limited proliferation capacity. ② Very short viable lifespan in vitro. ③ Potential cross-species translational barriers (if using animal cells). ④ Ethical constraints and limited availability.
**Established cell lines**	hCMEC/D3 LUHMES RBE4 bEnd.3 HUVEC	① Infinite lifespan and highly proliferative. ② Easy to culture, cost-effective, and highly reproducible across labs. ③ Bypasses the ethical constraints and supply limitations of primary human tissue.	① Prone to dedifferentiation and progressive loss of in vivo-like phenotypes. ② Frequently form a “leaky” barrier with significantly lower Transendothelial Electrical Resistance (TEER). ③ Altered expression of critical transporters and tight junction proteins.
**hiPSC-derived cells**	iPSC-BMEC NPCs HM HO HN	① Highly scalable. ② Capable of long-term maintenance of function and viability in vitro; overcomes interspecies differences.	① Prone to immature (fetal-like) phenotypes. ② Differentiation protocols are complex. ③ Costly, and time-consuming. ④ Inherent epigenetic variability and line-to-line variations.
**Tumor cells**	1321N1 SH-SY5Y PC12 C6	① Robust growth, easy maintenance, and infinite expansion capacity. ② Highly consistent phenotypic behavior across passages. ③ Specifically useful for modeling neuro-oncological microenvironments.	① Highly abnormal karyotypes and mutated genetic profiles. ② Fails to accurately recapitulate the healthy, physiological state of the NVU. ③ Exhibits inherently dysfunctional tight junctions and severely compromised barrier integrity.

**Table 3:** Hierarchical validation principles and methodologies for 3D NVU models.

Hierarchical Level	Key Objectives and Targeted Elements	Common Evaluation Methods
**Biological Integrity**	① Confirm completeness of cell composition. ② Spatial arrangement. Baseline neurotrophic effects.	① Cell viability assays. ② Morphological assessment ③ 3D spatial imaging of multicellular self-assembly.
**Physical Integrity**	① Assess the structural tightness and restrictiveness of the BBB.	① TEER measurements. ② Paracellular permeability assays (e.g., FITC-dextran, sodium fluorescein).
**Molecular Profiling**	① Verify the expression of specific cellular markers and TJPs (e.g., ZO-1, Claudin-5).	① Immunofluorescence staining. ② Reverse Transcription quantitative Polymerase Chain Reaction (RT-qPCR).
**Functional Responsiveness**	① Demonstrate physiological transport. ② Neurovascular coupling. ③ Secretion of specific neurotrophic factors.	① P-gp efflux transporter assays. ② Calcium imaging. ③ Enzyme-Linked Immunosorbent Assay (ELISA).

**Table 4:** Comparison of advantages and limitations of four main types of NVU models.

Type	Applicability	Advantage	Limitation	Reference
**Transwell Chamber NVU models**	① BBB ② Drug transport and screening ③ TEER monitoring	① Easy to construct ② High-throughput capability ③ Convenient sampling (dual chambers) ④ Improved 3D ECM microenvironment (with Matrigel)	① Single function ② Lack of 3D structure ③ Cannot simulate the real environment ④ Absence of shear stress	[84,88,89,98]
**Gel-PDMS-based NVU models**	① Simulated 3D structure ② Neurogenesis ③ Angiogenesis	① Structurally significant ② High degree of microenvironment simulation	① Cumbersome steps ② Many materials needed	[55,56,100,102,107,108,109,112,115]
**Self-assembled NVU models**	① Disease modeling ② Drug screening ③ Bioeffects study	① 3D BBB structure ② Multicellular interactions ③ Cost-effective and fast	① Structural heterogeneity ② Spherical geometry ③ 3D imaging challenges	[14,57,119]
**Microfluidic NVU models**	① BBB ② Metabonomics	① High throughput ② Separated regions	① Lack of 3D structure	[13,126,128]

## Data Availability

No new data were created or analyzed in this study. Data sharing is not applicable to this article.

## Supplementary Materials

The following supporting information can be downloaded at: https://www.mdpi.com/article/10.3390/biom16091250/s1, Table S1: Detailed characteristics of various in vitro NVU models.
